# Primate-specific evolution of noncoding element insertion into *PLA2G4C *and human preterm birth

**DOI:** 10.1186/1755-8794-3-62

**Published:** 2010-12-24

**Authors:** Jevon Plunkett, Scott Doniger, Thomas Morgan, Ritva Haataja, Mikko Hallman, Hilkka Puttonen, Ramkumar Menon, Edward Kuczynski, Errol Norwitz, Victoria Snegovskikh, Aarno Palotie, Leena Peltonen, Vineta Fellman, Emily A DeFranco, Bimal P Chaudhari, John Oates, Olivier Boutaud, Tracy L McGregor, Jude J McElroy, Kari Teramo, Ingrid Borecki, Justin C Fay, Louis J Muglia

**Affiliations:** 1 Department of Pediatrics, Vanderbilt University School of Medicine, Nashville, TN 37232, USA; 2 Human and Statistic Genetics Program, Washington University School of Medicine, St. Louis, MO 63110, USA; 3 Computational Biology Program, Washington University School of Medicine, St. Louis, MO 63108, USA; 4 Center for Human Genetics Research, Vanderbilt University School of Medicine, Nashville, TN 37232, USA; 5 Institute of Clinical Medicine, Department of Pediatrics, University of Oulu, Oulu 90014, Finland; 6 Departments of Obstetrics and Gynecology, University Central Hospital, Helsinki 00290, Finland; 7 The Perinatal Research Center, Nashville, TN 37203; 8 Department of Epidemiology, Rollins School of Public Health, Emory University, Atlanta, GA, 30322, USA; 9 Department of Obstetrics, Gynecology, and Reproductive Sciences, Yale University School of Medicine, New Haven, CT 06520, USA; 10 Biomedicum Helsinki Research Program in Molecular Medicine, University of Helsinki, Helsinki 00290, Finland; 11 The Finnish Genome Center, University of Helsinki, Helsinki 00290, Finland; 12 The Broad Institute of MIT and Harvard, Cambridge, Massachusetts 02142, USA; 13 Wellcome Trust Sanger Institute, Cambridge CB10 1SA, UK; 14 Department of Pediatrics, Lund University, Lund 22185, Sweden; 15 Department of Pediatrics, University of Helsinki, Helsinki 00290, Finland; 16 Department of Obstetrics and Gynecology, University of Cincinnati College of Medicine, Cincinnati, OH 45267, USA; 17 Department of Pediatrics, Washington University School of Medicine, St. Louis, MO 63110, USA; 18 Division of Clinical Pharmacology, Vanderbilt University School of Medicine, Vanderbilt University, Nashville, TN 37232, USA; 19 Division of Statistical Genomics, Washington University School of Medicine, St. Louis, MO 63108, USA; 20 Department of Genetics and Center for Genome Sciences, Washington University School of Medicine, St. Louis, MO 63108, USA; 21 Department of Molecular Physiology and Biophysics, Vanderbilt University School of Medicine, and Vanderbilt Kennedy Center for Human Development, Vanderbilt University, Nashville, TN 37232, USA

## Abstract

**Background:**

The onset of birth in humans, like other apes, differs from non-primate mammals in its endocrine physiology. We hypothesize that higher primate-specific gene evolution may lead to these differences and target genes involved in human preterm birth, an area of global health significance.

**Methods:**

We performed a comparative genomics screen of highly conserved noncoding elements and identified *PLA2G4C*, a phospholipase A isoform involved in prostaglandin biosynthesis as human accelerated. To examine whether this gene demonstrating primate-specific evolution was associated with birth timing, we genotyped and analyzed 8 common single nucleotide polymorphisms (SNPs) in *PLA2G4C *in US Hispanic (n = 73 preterm, 292 control), US White (n = 147 preterm, 157 control) and US Black (n = 79 preterm, 166 control) mothers.

**Results:**

Detailed structural and phylogenic analysis of *PLA2G4C *suggested a short genomic element within the gene duplicated from a paralogous highly conserved element on chromosome 1 specifically in primates. SNPs rs8110925 and rs2307276 in US Hispanics and rs11564620 in US Whites were significant after correcting for multiple tests (p < 0.006). Additionally, rs11564620 (Thr360Pro) was associated with increased metabolite levels of the prostaglandin thromboxane in healthy individuals (p = 0.02), suggesting this variant may affect *PLA2G4C *activity.

**Conclusions:**

Our findings suggest that variation in *PLA2G4C *may influence preterm birth risk by increasing levels of prostaglandins, which are known to regulate labor.

## Background

A growing body of evidence supports genetic influences on preterm birth risk; however, few genes have been consistently associated with the disorder [1,2]. Investigators have typically focused on candidate genes selected based on predicted parturition physiology; however, this approach may be limited by the divergence in physiological mechanisms between humans and model organisms that have been typically studied. For example, while a rapid decline in progesterone plays a prominent role in initiating parturition in rodents and sheep, this signal does not seem to precede human labor [3]. Other parturition-related traits, such as placental morphology and source of progesterone, also differ importantly in humans compared to model organisms typically studied and may limit what generalizations can be made [3].

Differences in parturition physiology between apes, including humans, and other mammals may have developed in response to uniquely human adaptations including relatively large human head size and narrow birth canal cross-sectional area [4]. Genes involved in parturition likely have evolved differentially along the human and/or higher primate phylogenetic lineages to decrease the length of gestation and alleviate the complications arising from such cephalopelvic constraints. As a result, the set of genes rapidly evolving on the human and/or higher primate lineage likely includes genes that play important roles in regulating parturition and potentially influence preterm birth risk. Consistent with our hypothesis, we identified *FSHR *as having rapidly evolved by nucleotide substitution and as being associated with preterm birth risk across independent populations ([5] and (Plunkett J, Doniger S, Orabona G, Morgan T, Haataja R, Hallman M, Puttonen H, Menon R, Kuczynski E, Norwitz E et al: Evolutionary history of *FSHR *in human predicts role in birth time, submitted)).

In addition to nucleotide substitution, genomic rearrangements account for a substantial portion of genomic divergence among species. For example, Frazer et al. [6] and Wetterbom et al. [7] observed insertions and deletions frequently when comparing genome sequences among humans, chimpanzees and other primate species. Moreover, such rearrangements may account for a larger fraction of genomic divergence than nucleotide substitutions [7]. Rearrangements can lead to loss or acquisition of exons, splice sites and promoters, facilitating differences in expression patterns, such as those observed for transcript variants of *CHRM3 *and *SFTPB *with differing transposable element insertion events [8,9]. Hence, genomic rearrangement may contribute to rapid evolution along the human and/or higher primate lineages in response to unique physiological constraints.

We hypothesized that genes with genomic rearrangements departing from the ancestral state and occurring on the human and/or higher primate lineages may play important roles in birth timing and preterm delivery. Thus, we investigated association with preterm birth for common variants in a gene, *PLA2G4C*, which is expressed in the uterus [10] and involved in prostaglandin synthesis, suggesting a potential role in parturition, and in which we have identified a primate-specific insertion.

## Results

### Evolutionary history of a primate-specific *PLA2G4C *noncoding element

**Figure 1:**
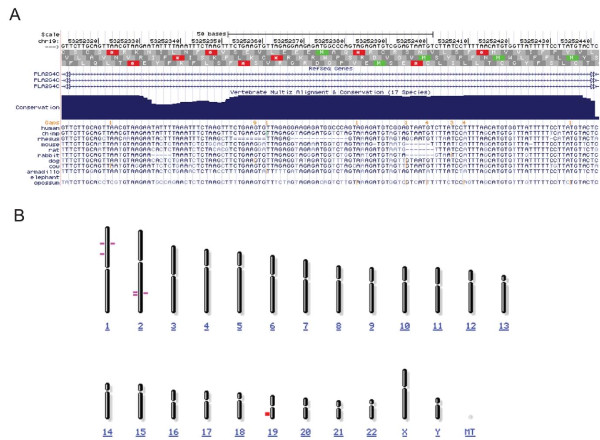
**Genomic alignments suggest *PLA2G4C *noncoding element duplicated from another chromosome**. MultiZ alignments used in the noncoding analysis from which we initially identified *PLA2G4C *as rapidly evolving include sequence for lower mammals, including cow, in which the gene is absent (Panel A). A BLASTN search of the element located in *PLA2G4C *intron 14 (red bar) (chromosome 19) that led to the gene's designation as rapidly evolving by nucleotide substitution revealed highly conserved noncoding elements (purple bars) on human chromosomes 1 and 2 (Panel B).

**Figure 2:**
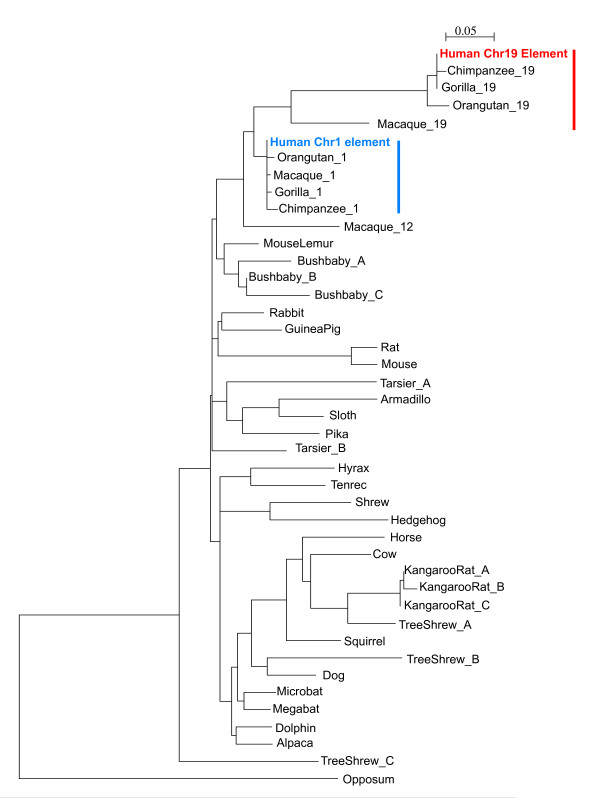
**Phylogeny with sequences homologous to human chromosomes 19 noncoding element**. Species name followed by a number indicates from which chromosome the sequence is derived or by a letter indicates that multiple copies homologous to the human chromosome 19 noncoding element were identified for that species. Sequences from lower primates and other mammalian species are more similar to higher primate sequences orthologous to human chromosome 1noncoding element (indicated in blue) than sequences orthologous to human chromosome 19 noncoding element (indicated in red). A duplication of chromosome 1 noncoding element to chromosome 19 likely occurred before the last common ancestor between apes and macaque.

**Figure 3:**
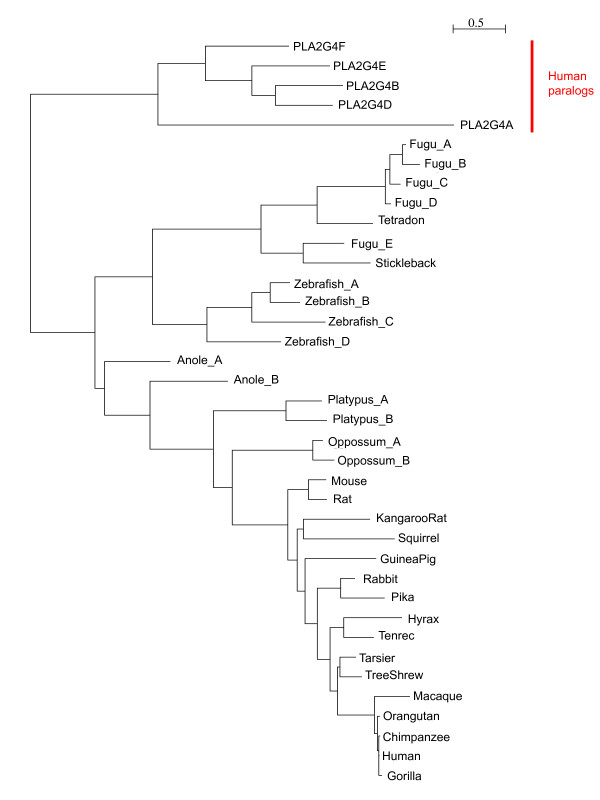
**Phylogeny with coding sequences homologous to human *PLA2G4C***. Species name followed by a letter indicates that multiple copies homologous to human *PLA2G4C *were identified for that species. Phylogenetic tree of coding sequences follows expected relationships between species, suggesting that the duplication event of chromosome 1 sequence to chromosome 19 did not include coding sequence.

We identified genes showing evidence of rapid evolution along the human lineage, based on evidence from a comparative genomic screen of highly conserved noncoding elements as previously described [5]. Among the rapidly evolving genes emerging from our noncoding screen, *PLA2G4C *was identified as the most statistically significant human-lineage accelerated gene (p = 2.2 × 10^-7^, significant at 10% False Discovery Rate threshold) that was also included in a list of preterm birth candidate genes [11]. Because the reported deletion of *PLA2G4C *in cattle [12] contrasted with its presence in the 17-way MultiZ alignments [13] used to identify the gene as rapidly evolving (Figure 1A), we examined the history of this region in greater depth. We compared sequence surrounding the 130 base pair (bp) highly conserved noncoding element in intron 14 of *PLA2G4C*, located on chromosome 19q13.3, which strongly suggested the gene's designation as rapidly evolving along the human lineage, in comparison to other mammalian and primate genomes. From such comparisons, we determined that this 130 bp element on human chromosome 19 was highly similar to a highly conserved noncoding element on human chromosome 1 (BLASTN 114/130 bp identical (87%), BLAST Expect value (i.e. number of matches of this similarity likely to occur by chance alone) = 5x10^-38^; Figure 1B). Subsequent analysis showed that the MultiZ alignments that we used in our comparative genomics screen had misaligned the human chromosome 19 element with sequences in other mammals which were orthologous to human chromosome 1. When appropriate alignments were examined, we observed that the human chromosome 19 element was nearly identical in higher primate species (chimpanzee, gorilla, orangutan, macaque) examined, but absent in syntenic sequences in lower primates (lemur, bushbaby, tarsier) and other mammalian species. Chromosome 1 elements from higher primates are more similar to lower primates and other mammalian species than chromosome 19 elements (Figure 2). The chromosome 1 element occurs in the 5' untranslated region of *RNF11*, a gene involved in inflammatory signaling (UniProt KB) in mouse. Thus, a duplication of chromosome 1 noncoding element to chromosome 19 likely occurred before the last common ancestor between apes and macaque. A phylogenetic tree of coding sequences for *PLA2G4C *follows the expected mammalian phylogeny (Figure 3), suggesting that the duplication did not include coding sequences. Together these results suggest that neither element would qualify as rapidly evolving along the human lineage due to nucleotide substitution, but the chromosome 19 element may represent a primate-specific change meriting further study.

### Association with preterm birth

**Figure 4:**
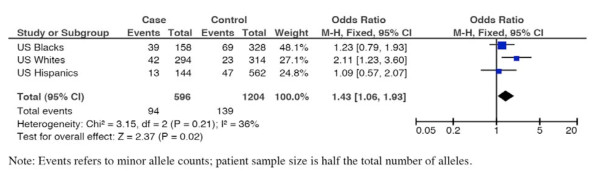
**Meta-analysis for US Hispanic, US White and US Black SNP association results for rs11564620**.

Having identified *PLA2G4C *as a candidate gene for regulation of parturition timing, we tested variants in this gene for association with preterm birth in a case-control study involving diverse clinical populations. Because of recent data suggesting that heritability of preterm birth risk acts largely or exclusively through the maternal genome [14-16], we genotyped US Hispanic (73 preterm, 292 control), US Whites (n = 147 preterm, 157 control) and US Black (n = 79 preterm, 166 control) mothers for 14 SNPs in the *PLA2G4C *gene region (Additional file 1 Table S1). We were able to analyze 8 of these 14 SNPs that met our quality and frequency cut-off criteria (Additional file 1 Table S1). Power analysis for allelic association in each of these relatively limited populations modeling a relative risk of 2.0 for the high risk allele showed actual power for detection at p < 0.05 (not correcting for multiple tests) ranging from 51-86%, and p < 0.006 (adjusting for 8 comparisons) ranging from 22 - 62% depending upon high risk allele frequency (Additional file 2 Table S2). The results from these analyses include two SNPs, rs8110925 and rs2307276, in the US Hispanics and one in the US Whites, rs11564620, that were significant after correcting for 8 tests (p < 0.006). Prior to constructing a meta-analysis, we performed a test for homogeneity across the three populations for each of these three SNPs. Two SNPs, rs8110925 and rs2307276, showed significant heterogeneity, implying population-specific effects. The heterogeneity p-value for rs11564620, in contrast, was not significant (p = 0.21), so we performed a formal meta-analysis across populations. In this circumstance, the test for overall effect across populations resulted in p = 0.02 (Figure 4). Given the relatively limited number of studies and subjects, we went on to gain additional biological support by measuring prostaglandin levels (see below).

Additionally, 2, 3 and 4 SNP haplotypes containing SNPs rs8110925 and rs2307276 were significant in the US Hispanics after correcting for 18 haplotype comparisons (p < 0.003), although not more significant than single SNP association findings (Additional file 3 Table S3, S4). 2 SNP haplotypes containing rs11564620 were moderately significant (p < 0.05) in US Whites (Additional file 3 Table S3, Additional file 4 Table S4). Linkage disequilibrium (LD) among SNPs rs8110925, rs2307276, and rs11564620 was very low (r^2 ^< 0.1) in the three populations studied (Additional file 5 Figure S1), suggesting multiple independent associations were observed.

### Association with prostaglandin concentrations

**Figure 5:**
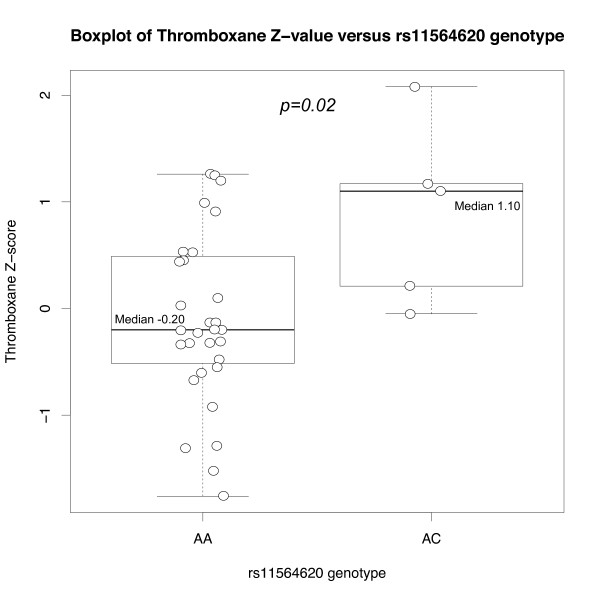
**Comparison of thromboxane metabolite levels among rs11564620 genotype classes in healthy control population**. Median thromboxane metabolite levels are significantly greater among risk-allele carriers, by Wilcoxon one-sided test.

To test the potential functional effect of associated *PLA2G4C *variants on prostaglandin metabolism, we compared levels of metabolites of prostaglandin E2 (PGE), prostaglandin I2 (PGI) and thromboxane (11-DTXB2) among genotype classes for associated SNPs rs8110925, rs2307276, and rs11564620 in healthy individuals using a two-sided Wald test (Additional file 6 Table S5). We hypothesized that these variants, particularly the coding region variant, would be associated with altered prostaglandin levels independent of pregnancy status. Of note, rs11564620, a nonsynonymous coding polymorphism, is associated with 11-DTXB2 levels (p = 0.04) despite the limited sample size available. The minor allele of rs11564620, present at approximately 10% frequency in US Whites, is associated with both risk for preterm birth and higher 11-DTXB2 levels (Wilcoxon one-sided p = 0.02; Figure 5).

## Discussion

Comparative genomic analysis is an attractive method for identifying genetic variation among species that may correlate with inter-specific phenotypic variation in fundamental processes such as parturition. Through such an analysis, we have identified a noncoding element in intron 14 of *PLA2G4C *on chromosome 19 representing a primate-specific change involving amplification and subsequent divergence but without increased nucleotide substitution. Having identified *PLA2G4C *as a candidate gene, we proposed that this duplicated element represents a primate-specific change with a potential regulatory role in human parturition.

**Figure 6:**
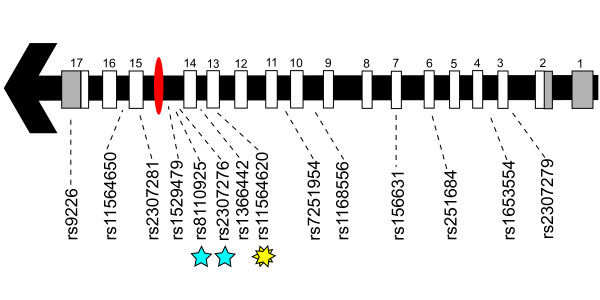
**Overview of the SNPs tested in the *PLA2G4C *gene region**. The gene structure for *PLA2G4C *is represented by an arrow in which grey rectangles designate 3' and 5' untranslated regions and white rectangles designate coding exons. A red ellipse represents the primate-specific element of interest. Blue stars indicate rs2307276 and rs8110925, and yellow starburst indicates rs11564620, which is significant after multiple testing correction (p < 0.006) in US Hispanics and US Whites, respectively.

We then tested our hypothesis by case-control association studies of preterm birth in several genetically diverse populations. Single SNP and haplotype association results implicated the role of SNPs rs8110925, rs2307276, and rs11564620 in preterm birth risk (Table 1 and Additional file 3 Table S3). The associated SNPs are located in an 8 kilobase (kb) region of the 3' end of *PLA2G4C*, near the genomic element of interest (Figure 6), but show little LD with each other (Additional file 5 Figure S1) or other SNPs in *PLA2G4C *documented in the International HapMap Project database [17]. Of note, Polyphen [18] and SIFT [19] programs predict rs11564620, a nonsynonymous polymorphism in exon 13 resulting in a change in amino acid 360 from threonine to proline, to be possibly damaging to the protein structure (using protein sequence NP_003697 for PLA2G4C). This 8 kb region also includes coding sequence for aspartic acid 385, one of the three amino acids that make up the putative active site of the enzyme [20], such that the proline substitution may alter the active site's physical conformation. Supporting the potential functional effect of rs11564620, this polymorphism is associated with 11-DTXB2 levels in healthy individuals (p = 0.02; Figure 5), with proline allele carriers having elevated thromboxane metabolite levels, compared to threonine homozygotes. Further suggesting functional importance of rs11564620, this SNP is also significantly associated (p < 0.0001) with altered expression of NFATC2IP, a factor regulating cytokine expression in T cells located on chromosome 16 in quantitative trait databases for CEU populations http://scan.bsd.uchicago.edu/newinterface/about.html. Last, the evidence of modest deviation (p = 0.04) from Hardy-Weinberg Equilibrium in the control US White population suggests that this variant may be under selection consistent with our hypothesis of selective pressure on genes involved in human parturition.

**Table 1:** Case-control association results for significant and suggestive SNPs in the PLA2G4C gene region tested across 3 independent populations.

Population	SNP	Allele	Case Frequency	Control Frequency	Allele p-value	Genotype p-value	Allelic OR (95% CI)
US Hispanic (73 cases, 292 controls)	rs8110925	G	0.18	0.085	**7.92 × 10^-4 a, b^**	**5.66 × 10^-5 b^**	2.4 (1.4-4.1)
	
	rs2307276	A	0.11	0.036	**5.45 × 10^-3 b^**	0.01	3.2 (1.6-6.5)
	
	rs1366442	A	0.49	0.36	**0.01**	**0.03**	1.7 (1.2-2.4)
	
	rs11564620	G	0.09	0.08	0.55 ^c^	0.63 ^c^	1.1 (0.6-2.1)

							

US White (147 cases, 157 controls)	rs8110925	G	0.058	0.057	0.92	0.59	1.0 (0.5-2.0)
	
	rs2307276	A	0.031	0.041	0.87	0.86	0.8 (0.3-1.8)
	
	rs1366442	G	0.4	0.42	0.5	**0.02**	0.9 (0.6-1.2)
	
	rs11564620^d^	G	0.14	0.07	**6.98 × 10^-3^**	**1.03 × 10^-3 b^**	2.1 (1.2-3.6)

							

US Black (79 cases, 166 controls)	rs8110925	G	0.22	0.21	0.58 ^e^	0.83 ^e^	1.1 (0.7-1.7)
	
	rs2307276	A	0.093	0.14	0.22	0.49	0.6 (0.3-1.2)
	
	rs1366442	G	0.42	0.4	0.92	0.94	1.1 (0.7-1.6)
	
	rs11564620	G	0.25	0.21	0.47 ^c^	0.36 ^c^	1.2 (0.8-1.9)

^a^Bolded numbers indicate p-value <0.05.

^b^Marker significant correcting for 8 tests (p < 0.006).

^c^Same allele trends in same direction as US Whites.

^d^p = 0.04 for deviation from Hardy-Weinberg Equilibrium.

^e^Same allele trends in same direction as US Hispanics.

*PLA2G4C *encodes cytosolic phospholipase A2 gamma, which hydrolizes phospholipids from the cellular membrane to form free arachidonic acid, from which prostaglandins, including prostaglandins D, E, F, I2 (also known as prostacyclin), and thromboxane A2 are generated. Prostaglandins play an important role in parturition. Pharmacologically, prostaglandins are used to induce abortion, for cervical ripening, and labor induction and drugs inhibiting prostaglandin synthesis are successful in preventing preterm labor [21]. Levels of prostaglandins, including thromboxane A2, are elevated in pregnant compared to non-pregnant women, and in late (36 weeks) compared to early (20, 30 weeks) gestation [22], suggesting a link between prostaglandin abundance and parturition timing. Prostaglandins may facilitate labor by several mechanisms. These hormones are known uterotonic agents and also promote luteolysis and the onset of labor in species that exhibit progesterone withdrawal prior to birth [23]. Prostaglandins may also facilitate delivery by affecting placenta function, since thromboxane A2 induces platelet aggregation and acts as a vasoconstrictor [22]. Hence, higher prostaglandin levels than expected may initiate parturition prematurely and lead to preterm delivery.

The PLA2G4C enzyme is the only cytosolic phospholipase A2 family member that is constitutively associated with the cellular membrane, the site of prostaglandin synthesis, rather than translocating to the membrane in response to calcium signaling [10]. Hence, dysregulation of *PLA2G4C *may alter prostaglandins levels independent of other parturition signals, such as oxytocin [24], that act via intracellular calcium signaling. For example, rs11564620 may contribute to a conformational change in the enzyme's active site, rendering it more active than usual and leading to increased synthesis of prostaglandins, as demonstrated by our observation of higher levels of thromboxane A2 in minor allele carriers for this polymorphism. Moreover, multiple splice isoforms of *PLA2G4C *exist, differing in transcript length, presence of certain exons and overlapping exons with different boundaries (AceView, NCBI, http://www.ncbi.nlm.nih.gov/IEB/Research/Acembly/). As a result, variation in *PLA2G4C *may contribute to differences in tissue-specific expression or relative abundance of various *PLA2G4C *isoforms, potentially altering function. Further study of the region encompassing these SNPs, including the genomic element of interest, is needed to examine the mechanism by which variation in *PLA2G4C *influences birth timing.

Specialization within multi-gene families, like the large phospholipase A2 gene family, can create individualized functions among paralogous genes. For example, *PLA2G4C *has a continuous association with the cellular membrane, unlike other phospholipase A2 genes, potentially differentiating its role in prostaglandin synthesis from those of other family members. Genomic variation, such as the element insertion observed in *PLA2G4C*, may contribute to gene specialization, as demonstrated by divergence in *PLA2G4C *expression patterns in humans versus mice, who lack the element insertion and express *PLA2G4C *only in ovary and oocytes[25]. Specialized genes are potentially better therapeutic targets than gene products with multiple roles within cell, since pharmaceutically targeting such genes may lead to fewer side effects. As a result, *PLA2G4C *may be a useful target for designing novel therapies to prolong pregnancy and reduce the incidence of preterm birth.

## Conclusions

A higher primate-specific noncoding element insertion into intron 14 of the phospholipase A2 gene *PLA2G4C *was identified, demonstrating the gene's rapid evolution along the higher primate lineage by genomic rearrangement. Results from our genetic analysis suggest common variation in *PLA2G4C *influences preterm birth risk. One of the variants associated with preterm birth is a nonsynonymous coding polymorphism, rs11564620, predicted to be potentially altering protein structure. This coding polymorphism is also associated with thromboxane levels, suggesting that genetic variation in *PLA2G4C *may increase risk for preterm birth by increasing levels of prostaglandins, which are known to regulate labor. By examining rapid evolution along human and higher primate lineages by genomic rearrangement, we have identified a novel gene associated with preterm birth. This approach can be readily applied to other traits differing among humans and/or higher primates and other species to aid in gene discovery.

## Methods

### Genomic alignments to investigate evolutionary history of *PLA2G4C*

Noting the deletion of *PLA2G4C *reported in the Taurine Cattle genome [12] contrasted with its presence in the 17-way MultiZ alignments [13] we used to identify the gene as rapidly evolving (analysis conducted Spring 2007 and presented in detail in (Plunkett J, Doniger S, Orabona G, Morgan T, Haataja R, Hallman M, Puttonen H, Menon R, Kuczynski E, Norwitz E et al: Evolutionary history of *FSHR *in human predicts role in birth time, submitted)), we examined the history of this region in greater depth. We extracted sequence surrounding the 130 bp highly conserved noncoding element (human chromosome 19: 48,560,500 -48,560,630; hg19 genome build) which largely contributed to our designation of *PLA2G4C *as rapid evolving along the human lineage. A BLASTN search of the element revealed highly identical conserved noncoding elements on human chromosomes 1 (87% identity) and 2 (85% identity) (Figure 1B). We compared the human chromosome 19 and chromosome 1 sequences to 31 eutherian mammalian genomes using Ensembl Genomic alignments (accessed September 2009), and ClustalW alignment, and to specific primate genomes using BLASTN searches of human, chimpanzee, gorilla, orangutan, macaque, and bushbaby genomes (accessed September 2009). We then reconstructed history of the element by creating phylogenies using maximum likelihood with sequences homologous to the human chromosome 19 element (Figure 2) and coding sequences homologous to human *PLA2G4C *(Figure 3).

### Human subjects

Study subjects were enrolled for genetic analysis by methods approved by Institutional Review Boards/Ethics Committees at each participating institution. Informed consent was obtained for all participants. Mothers with preterm birth were included if the birth was spontaneous (non-iatrogenic), singleton, had no obvious precipitating stimulus (trauma, infection, drug use), and was less than 37 weeks (Yale University; New York University) or 36 weeks (Centennial Hospital, Nashville, TN) of completed gestation. Control mothers were included if they had delivered two or more children at 37 weeks or later spontaneously. Healthy volunteers were recruited at Vanderbilt University for studies of prostaglandin metabolism. DNA from blood or saliva was prepared by standard methods. Race/ethnicity was assigned by self-report. All specimens were linked with demographic and medical data abstracted from maternal/neonatal records. DNA from blood or saliva was prepared by standard methods. Maternal age did not differ between cases and controls in the different populations (27.3 y vs. 28.4 y, p = 0.10 US White; 25.3 y vs. 25.2 y, p = 0.88 US Black; 26.0 y vs. 25.0 y, p = 0.20 US Hispanic).

### Prostaglandin metabolite levels

For individuals enrolled in the prostaglandin study, urine was collected by standard methods. Levels of the urinary metabolites of prostaglandin E (PGE), prostaglandin I (PGI) and thromboxane (11-DTXB2) were quantified by mass spectrometry and normalized to creatinine levels, an indicator of renal function, in 44 healthy control individuals of Black, Hispanic or White race (median age 29, 60% male, 77% White).

### Genotyping

We genotyped 14 SNPs spanning the *PLA2G4C *gene region (Additional file 1 Table S1) on human chromosome 19 in cohorts of US Hispanics (n = 73 preterm, 292 control mothers), US Whites (n = 147 preterm, 157 control mothers) and US Blacks (n = 79 preterm, 166 control mothers). For SNP selection, data from the HapMap Release 27 CEU population was examined in the Haploview program [26], using tagger and haplotype block functions, to identify regions of high LD. We selected 1 SNP per haplotype block, defined using the D' confidence interval method [27], having the highest minor allele frequency (MAF) in the CEU population for genotyping. We also included coding SNPs and other noncoding SNPs to improve coverage of conserved elements contributing to the gene's designation as "rapidly evolving." This selection scheme resulted in approximately 35% coverage of the gene region at r^2^≥0.8. SNPs showing evidence of association in one or more cohort (p < 0.01; n = 4) were then genotyped in healthy individuals on whom data on their concentrations of several prostaglandin metabolites was available to examine potential functional effects of the variants. All SNPs were genotyped using the Sequenom iPLEX massARRAY technology (Sequenom, San Diego, CA).

### Data Analysis

Data cleaning and analysis was performed with Whole-genome Association Study Pipeline (WASP) [28] and PLINK [29]. We excluded individuals based on genotyping quality (< 90% call rate) and SNPs based on the following criteria: not in Hardy-Weinberg Equilibrium in controls (p < 0.001 χ^2 ^test), <90% genotype call rate, MAF < 0.01). Linkage disequilibrium among SNPs tested was determined using the Haploview program [26]. We chose this Hardy-Weinberg threshold for two reasons. First, we hypothesize this locus is under selective pressure which could result in some deviation from HWE in the control population. Second, samples with p < 0.001 also had low genotype call rates, suggesting genotyping error, while those with p > 0.001 had high call rates. For significant SNPs, the Hardy-Weinberg deviation was greater than 0.05 unless otherwise indicated. We corrected for multiple testing using the simpleM method [30], which estimates the number of independent tests, given the LD relationships among SNPs, used to obtain a Bonferroni-corrected critical value.

Our analysis considered preterm birth affection status (i.e. delivery <37 weeks) as a binary trait, comparing frequencies between case and control groups of alleles and genotypes by χ^2 ^test. Sliding windows of 2,3 and 4 SNP haplotypes also were compared between cases and controls [29]. Meta-analysis of data for significant SNPs was done using the Mantel-Haenszel method, after successfully passing the test of homogeneity.

To test the potential functional effect of associated *PLA2G4C *variants on prostaglandin metabolism, we examined the levels of PGE, PGI, and 11-DTXB2, standardized to normal distributions (μ = 0, σ = 1), as quantitative traits. A Wald test was performed to compare the mean phenotype between different allele or genotype classes for associated SNPs. We also tested whether rs11564620 risk-allele carriers had higher prostaglandin levels than noncarriers, by comparing the 11-DTXB2 value distribution among genotype classes with box plots and one-sided Wilcoxon nonparametric test performed in R [31].

## Authors' contributions

JP, SD, TM, JO, OB, JF and LM designed research; RH, MH, HP, RM, EK, EN, VS, AP, LP, VF, ED, BC, JO, OB, KT and LJM contributed new reagents/analytic tools; JP, SD, GO performed research; JP, SD, JJM, TLM, TM, OB, JF and LJM analyzed data; and JP, MH, EO, JO, KT, IB, JF, TM, and LJM wrote the paper. All authors read and approved the final manuscript.

## Pre-publication history

The pre-publication history for this paper can be accessed here:

http://www.biomedcentral.com/1755-8794/3/62/prepub

## Supplementary Material

Additional file 1 **SNPs in the *PLA2G4C *gene region tested in all cohorts**. Table S1 - SNPs examined in our association study. Click here for file

Additional file 2 **Power analysis for populations analyzed in this study for association with preterm birth risk**. Table S2 - Power analyses for the populations tested for preterm birth risk. Click here for file

Additional file 3 **Case-control association results for 2, 3 and 4 SNP haplotypes in the *PLA2G4C *gene region tested across 3 independent US populations**. Table S3 - Case-control association results for 2, 3 and 4 SNP haplotypes in the *PLA2G4C *gene region. Click here for file

Additional file 4 **Frequency of 2, 3, and 4 SNP haplotypes across each population studied**. Table S4 Specific frequency information for the haplotypes in each population. Click here for file

Additional file 5 **Linkage disequilibrium among SNPs tested in *PLA2G4C***. Figure S1 - Panel A: US Hispanics. Panel B: US Whites. Panel C: US Blacks. Panel D: HapMap MEX reference population. Panel E: HapMap CEU reference population. Panel F: HapMap YRI reference population. Click here for file

Additional file 6 **Association results for associated SNPs (p ≤ 0.01) in the *PLA2G4C *gene region for the quantitative phenotypes of PGE, PGI, and TXB2 metabolite levels examined in healthy individuals (n = 44)**. Table S5 Association results for those SNPs that were significantly associated (p ≤ 0.01) in the *PLA2G4C *gene region with preterm birth examining the quantitative phenotypes of prostaglandin metabolite levels examined in healthy individuals. Click here for file
