# Supplementary material for: Primate-specific evolution of noncoding element insertion into PLA2G4C and human preterm birth
Source: BMC Med Genomics. 2010 Dec 24;3:62. doi: 10.1186/1755-8794-3-62 (PMC3017005; doi:10.1186/1755-8794-3-62)
Supplement: Additional file 1 — SNPs in the PLA2G4C gene region tested in all cohorts. Table S1 - SNPs examined in our association study. [file 1755-8794-3-62-S1.DOCX]

**Table S1: SNPs in the *PLA2G4C* gene region tested in all cohorts.**

| SNP | Position ^a^ | Location within gene |
| --- | --- | --- |
| rs9226^b,c^ | 48,551,546 | Exon 17 - 3' UTR |
| rs11564650^e^ | 48,556,979 | Intron 15 |
| rs2307281^a^ | 48,558,286 | Exon 15 - Asp426Asp |
| rs1529479^c^ | 48,558,388 | Intron 14 |
| rs8110925 | 48,563,432 | Intron 14 |
| rs2307276 | 48,565,207 | Intron 14 |
| rs1366442 | 48,569,709 | Intron 13 |
| rs11564620 | 48,571,072 | Exon 13 - Thr360Pro |
| rs7251954^b,c^ | 48,583,239 | Intron 10 |
| rs11668556^b,c^ | 48,591,380 | Intron 9 |
| rs156631^b,d^ | 48,598,823 | Exon 7- Ser203Pro |
| rs251684^b^ | 48,601,454 | Exon 6 - Pro170Pro |
| rs1653554^e^ | 48,608,472 | Intron 3 |
| rs2307279^b,c^ | 48,608,598 | Exon 3 - Ala38Pro |

^a^ Positions refer to hg19 build of the human genome.

^b^ Marker excluded because of <90% call rate.

^c^ Marker excluded because of Hardy-Weinberg Equilibrium failure in controls p<0.001.

^d^ Marker excluded because of MAF<0.01.

^e^ Non-significant p value (>0.10) in each population.
