# Supplementary material for: Primate-specific evolution of noncoding element insertion into PLA2G4C and human preterm birth
Source: BMC Med Genomics. 2010 Dec 24;3:62. doi: 10.1186/1755-8794-3-62 (PMC3017005; doi:10.1186/1755-8794-3-62)
Supplement: Additional file 2 — Power analysis for populations analyzed in this study for association with preterm birth risk. Table S2 - Power analyses for the populations tested for preterm birth risk. [file 1755-8794-3-62-S2.PDF]

Table S2: Power analysis for allelic case-control association in US Hispanics, US White, and US Black populations tested. A relative risk for the high risk genotypes of 2.0 (AA = Aa) is modeled. Calculations were done in Genetic Power Calculator (http://pngu.mgh.harvard.edu/~purcell/gpc/).

High Risk allele freq	Prevalence	Genotype relative risk	D'	Marker allele freq	# cases	Ratio control: cases	Type I error (single SNP)	Actual Power (single SNP)	Type I error (8 tests)	Actual Power (8 tests)
US Hispanic										
0.05	0.1	2.0, 2.0	1	0.05	73	4	0.05	0.58	0.006	0.28
0.1	0.1	2.0, 2.0	1	0.1	73	4	0.05	0.75	0.006	0.46
0.2	0.1	2.0, 2.0	1	0.2	73	4	0.05	0.79	0.006	0.51
0.3	0.1	2.0, 2.0	1	0.3	73	4	0.05	0.71	0.006	0.41
0.4	0.1	2.0, 2.0	1	0.4	73	4	0.05	0.57	0.006	0.27
US Black										
0.05	0.1	2.0, 2.0	1	0.05	79	2.1	0.05	0.51	0.006	0.22
0.1	0.1	2.0, 2.0	1	0.1	79	2.1	0.05	0.68	0.006	0.38
0.2	0.1	2.0, 2.0	1	0.2	79	2.1	0.05	0.74	0.006	0.44
0.3	0.1	2.0, 2.0	1	0.3	79	2.1	0.05	0.67	0.006	0.36
0.4	0.1	2.0, 2.0	1	0.4	79	2.1	0.05	0.54	0.006	0.24
US White										
0.05	0.1	2.0, 2.0	1	0.05	147	1.1	0.05	0.62	0.006	0.31
0.1	0.1	2.0, 2.0	1	0.1	147	1.1	0.05	0.8	0.006	0.52
0.2	0.1	2.0, 2.0	1	0.2	147	1.1	0.05	0.86	0.006	0.62
0.3	0.1	2.0, 2.0	1	0.3	147	1.1	0.05	0.81	0.006	0.53
0.4	0.1	2.0, 2.0	1	0.4	147	1.1	0.05	0.68	0.006	0.38
