# Supplementary material for: Primate-specific evolution of noncoding element insertion into PLA2G4C and human preterm birth
Source: BMC Med Genomics. 2010 Dec 24;3:62. doi: 10.1186/1755-8794-3-62 (PMC3017005; doi:10.1186/1755-8794-3-62)
Supplement: Additional file 3 — Case-control association results for 2, 3 and 4 SNP haplotypes in the PLA2G4C gene region tested across 3 independent US populations. Table S3 - Case-control association results for 2, 3 and 4 SNP haplotypes in the PLA2G4C gene region. [file 1755-8794-3-62-S3.DOCX]

**Table S3: Case-control association results for 2, 3 and 4 SNP haplotypes in the *PLA2G4C* gene region tested across 3 independent US populations.**

|  | US Hispanics | US White | US Blacks |
| --- | --- | --- | --- |
|  | p-value | p-value | p-value |
| *2-SNP Haplotypes* | | | |
| rs11564650-rs8110925 | **9.54x10^-3 a^** | 0.10 | 0.59 |
| rs8110925-rs2307276 | **6.98x10^-5 b^** | 0.98 | 0.24 |
| rs2307276-rs1366442 | **8.82x10^-3^** | 0.64 | 0.64 |
| rs1366442-rs11564620 | **0.04** | **0.03** | 0.90 |
| rs11564620-rs11668556 | 0.25 | **0.03** | 0.63 |
| rs11668556-rs1653554 | 0.49 | 0.82 | 0.42 |
| rs1653554-rs2307279 | NA^c^ | 0.47 | 0.68 |
| *3-SNP Haplotypes* | | | |
| rs11564650-rs8110925-rs2307276 | **9.60x10^-4 b^** | 0.19 | 0.39 |
| rs8110925-rs2307276-rs1366442 | **5.79x10^-4 b^** | 0.84 | 0.50 |
| rs2307276-rs1366442-rs11564620 | **0.03** | 0.06 | 0.79 |
| rs1366442-rs11564620-rs11668556 | 0.06 | 0.08 | 0.92 |
| rs11564620-rs11668556-rs1653554 | 0.31 | 0.09 | 0.30 |
| rs11668556-rs1653554-rs2307279 | NA^c^ | 0.91 | 0.89 |
| *4-SNP Haplotypes* | | | |
| rs11564650-rs8110925-rs2307276-rs1366442 | **3.26x10^-3^** | 0.24 | 0.67 |
| rs8110925-rs2307276-rs1366442-rs11564620 | **1.25x10^-3 b^** | 0.13 | 0.46 |
| rs2307276-rs1366442-rs11564620-rs11668556 | 0.08 | 0.17 | 0.93 |
| rs1366442-rs11564620-rs11668556-rs1653554 | 0.08 | 0.25 | 0.47 |
| rs11564620-rs11668556-rs1653554-rs2307279 | NA^c^ | 0.11 | 0.54 |

^a^Bolded numbers indicate p-value <0.05.

^b^Haplotype significant correcting for 18 comparisons (p< 2.78x10^‑3^).

^c^ One or more marker excluded for failing one or more of the following measures: Hardy-Weinberg Equilibrium failure in controls p<0.001, <90% call rate, MAF<0.01.
