# Supplementary figures and images for: Primate-specific evolution of noncoding element insertion into PLA2G4C and human preterm birth
Source: BMC Med Genomics. 2010 Dec 24;3:62. doi: 10.1186/1755-8794-3-62 (PMC3017005; doi:10.1186/1755-8794-3-62)

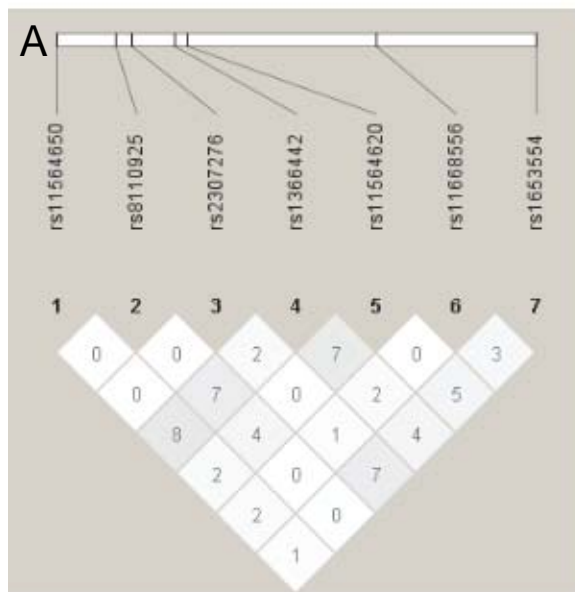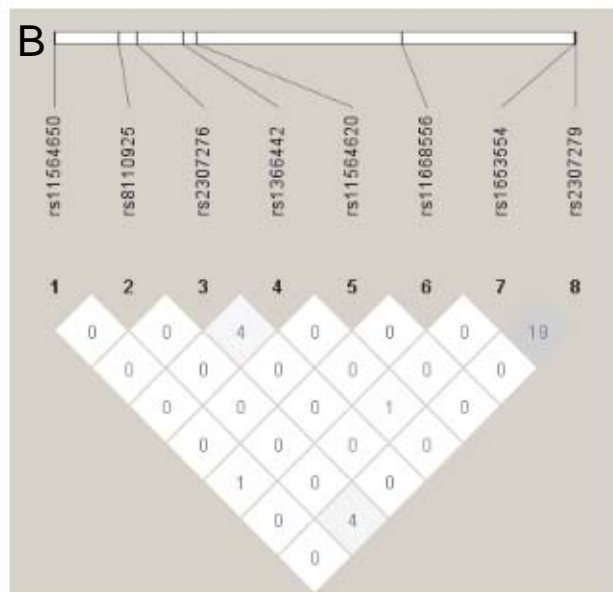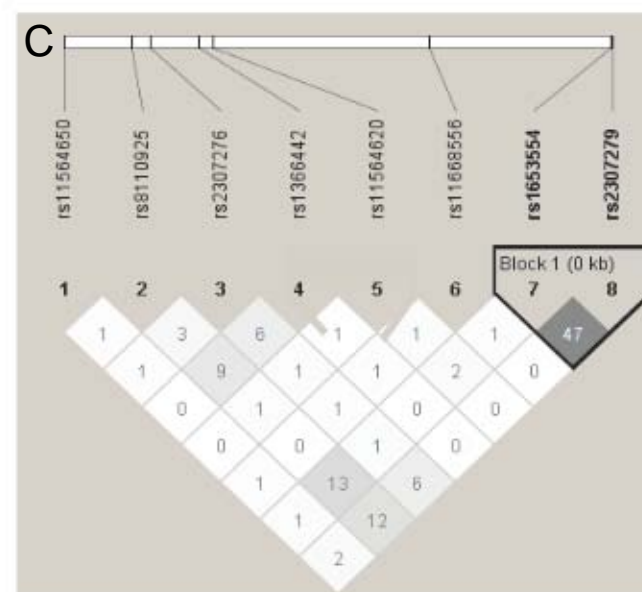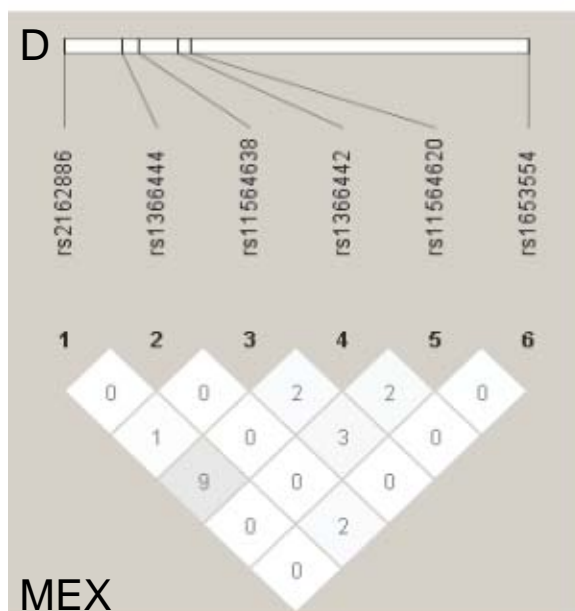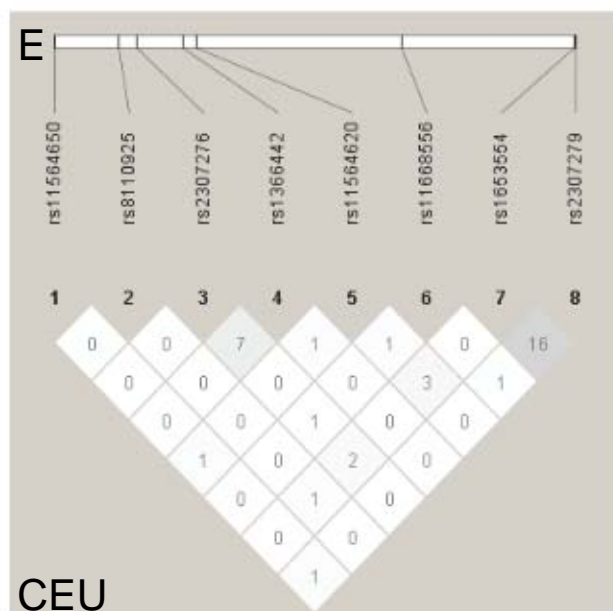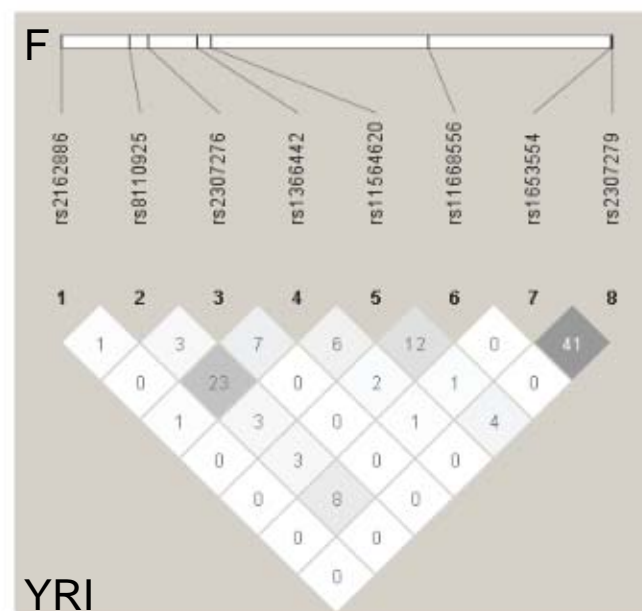

Supplement: Additional file 5 — Linkage disequilibrium among SNPs tested in PLA2G4C. Figure S1 - Panel A: US Hispanics. Panel B: US Whites. Panel C: US Blacks. Panel D: HapMap MEX reference population. Panel E: HapMap CEU reference population. Panel F: HapMap YRI reference population. [file 1755-8794-3-62-S5.PDF]
