# Supplementary material for: Primate-specific evolution of noncoding element insertion into PLA2G4C and human preterm birth
Source: BMC Med Genomics. 2010 Dec 24;3:62. doi: 10.1186/1755-8794-3-62 (PMC3017005; doi:10.1186/1755-8794-3-62)
Supplement: Additional file 6 — Association results for associated SNPs (p ≤ 0.01) in the PLA2G4C gene region for the quantitative phenotypes of PGE, PGI, and TXB2 metabolite levels examined in healthy individuals (n = 44). Table S5 Association results for those SNPs that were significantly associated (p ≤ 0.01) in the PLA2G4C gene region with preterm birth examining the quantitative phenotypes of prostaglandin metabolite levels examined in healthy individuals. [file 1755-8794-3-62-S6.PDF]

Table S5: Association results for associated SNPs (p≤0.01) in the *PLA2G4C* gene region for the quantitative phenotypes of PGE, PGI, and TXB2 metabolite levels examined in healthy individuals (n=44).

SNP	PGE p-value	PGI p-value	TXB2 p-value
rs8110925	0.44	0.42	0.51
rs2307276	0.50	0.75	0.80
rs11564620	0.57	0.15	**0.04**[a]

[a]Bolded numbers indicate p-value <0.05.
